# Trauma exposure interacts with impulsivity in predicting emotion regulation and depressive mood

**DOI:** 10.3402/ejpt.v5.24104

**Published:** 2014-09-29

**Authors:** Grazia Ceschi, Joël Billieux, Melissa Hearn, Guillaume Fürst, Martial Van der Linden

**Affiliations:** 1 Clinical Psychology, University of Geneva, Geneva, Switzerland; 2 Laboratory for Experimental Psychopathology, Psychological Science Research Institute, Université catholique de Louvain, Louvain-la-Neuve, Belgium

**Keywords:** Trauma, impulsivity, cognitive emotion regulation, well-being, depressive mood, UPPS

## Abstract

**Background:**

Traumatic exposure may modulate the expression of impulsive behavioral dispositions and change the implementation of emotion regulation strategies associated with depressive mood. Past studies resulted in only limited comprehension of these relationships, especially because they failed to consider impulsivity as a multifactorial construct.

**Objective:**

Based on Whiteside and Lynam's multidimensional model that identifies four distinct dispositional facets of impulsive-like behaviors, namely urgency, (lack of) premeditation, (lack of) perseverance, and sensation seeking (UPPS), the current study used a sample of community volunteers to investigate whether an interaction exists between impulsivity facets and lifetime trauma exposure in predicting cognitive emotion regulation and depressive mood.

**Methods:**

Ninety-three adults completed questionnaires measuring lifetime trauma exposure, impulsivity, cognitive emotion regulation, and depressive mood.

**Results:**

Results showed that trauma-exposed participants with a strong disposition toward urgency (predisposition to act rashly in intense emotional contexts) tended to use fewer appropriate cognitive emotion regulation strategies than other individuals. Unexpectedly, participants lacking in perseverance (predisposition to have difficulties concentrating on demanding tasks) used more appropriate emotion regulation strategies if they had experienced traumatic events during their life than if they had not. Emotion regulation mediated the path between these two impulsivity facets and depressive mood.

**Conclusions:**

Together, these findings suggest that impulsivity has a differential impact on emotion regulation and depressive mood depending on lifetime exposure to environmental factors, especially traumatic events.

There is strong evidence that emotional distress after trauma varies in its frequency, severity, and quality (Brewin, 2003). Currently, it is widely acknowledged that short- and long-term posttraumatic outcomes may extend far beyond clinical posttraumatic stress disorder (PTSD; Ceschi, Hearn, Billieux, & Van der Linden, 2011). Indeed, depressive mood has often been observed after trauma exposure (David, Ceschi, Billieux, & Van der Linden, 2008). So far, however, relatively few studies have investigated a potential cognitive modulator or mediator that might account for the differential impact of traumatic experiences on subsequent depressive mood.

Recent studies in non-clinical samples have identified maladaptive emotion regulation strategies as possible correlates in the relationship between traumatic exposure and different kinds of general distress, including depressive mood (Martin & Dahlen, 2005; Tull, Barrett, McMillan, & Roemer, 2007). Research has shown that cognitive emotion regulation strategies are crucial for adaptation to stressful life events (Ochsner & Gross, 2005). Despite some inconsistent findings, cognitive emotion regulation strategies such as self-blame, catastrophizing, rumination, and reappraisal have consistently been shown to play an important role in depression (Garnefski & Kraaij, 2006; Garnefski, Teerds, Kraaij, Legerstee, & van den Kommer, 2004), as well as in the relationship between negative life events and depressive symptoms (Garnefski, Kraaij, & Spinhoven, 2001). Indeed, the association between cognitive emotion regulation (especially inappropriate coping strategies) and depression has repeatedly been demonstrated in trauma-exposed participants, including stalking victims (Kraaij, Arensman, Garnefski, & Kremers, 2007), adolescents with chronic disease (Garnefski, Koopman, Kraaij, & ten Cate, 2009), and children living in combat zones (Amone-P'Olak, Garnefski, & Kraaij, 2007). Furthermore, different cognitive emotion regulation strategies have been identified as mediators in the relationship between trauma and emotional distress (childhood abuse and depression: Raes & Hermans, 2008; violence and social adjustment: Schwartz & Proctor, 2000), and a moderator in the relationship between stressful life events and resilience (Troy & Mauss, 2011). These findings suggest that cognitive emotion regulation may be a powerful factor explaining the relationship between trauma and general emotional distress, including depressive mood. This might be especially relevant for individuals whose personal dispositions lead them to implement inappropriate cognitive emotion regulation strategies. Previous studies have identified impulsivity as a personal disposition that underlies significant interindividual differences in cognitive emotion regulation.

It has been revealed that, after adverse events exposure, impulsivity is associated with more severe PTSD (Fehon, Grilo, & Lipschitz, 2005) and depression (Brodsky et al., 2001). Conversely, trauma exposure tends to increase impulsivity (Braquehais, Oquendo, Baca-García, & Sher, 2010), which then increases the danger of subsequent exposure to trauma, especially due to risk-taking behaviors (Ben-Zur & Zeidner, 2009) and self-harm (Jallade, Sarfati, & Hardy-Baylé, 2005). For instance, a recent study conducted on 1,265 prisoners showed that individuals reporting a high-impulsivity disposition engaged in other- and self-aggressive behaviors more frequently, possibly resulting in further traumatic experiences (Carli et al., 2010). Although contradictory findings have also been published (Bevilacqua et al., 2012), together, these results suggest that trauma and impulsivity may indeed exert a multiplier effect on interindividual cognitive emotion regulation differences.

Previous studies established a clear link between impulsivity and emotional reactions after trauma exposure in clinical (abuse survivors: Brodsky et al., 2001; accident survivors: Joseph, Dalgleish, Thrasher, & Yule, 1997) and non-clinical samples alike (students: Aidman & Kollaras-Mitsinikos, 2006). However, as reviewed by Simmen-Janevska, Brandstätter, and Maercker (2012), these studies result in only limited comprehension of this relationship as they failed to consider impulsivity as a multifactorial construct. Indeed, research in the past decade has deconstructed impulsivity by showing that it encompasses a combination of distinguishable dimensions linked to specific cognitive and motivational mechanisms (Billieux, Gay, Rochat, & Van der Linden, 2010).

The urgency, (lack of) perseverance, (lack of) premeditation, and sensation seeking (UPPS) model of impulsivity (Whiteside & Lynam, 2001) was a noteworthy attempt to delimit the components of impulsive behaviors. Whiteside and Lynam (2001) administered the main recognized scales assessing impulsivity-related constructs to a large sample of participants, which led to the identification of four stable impulsivity components. These components are defined as follows: (1) Urgency, the tendency to act rashly when experiencing intense emotions; (2) (lack of) Premeditation, the tendency to ignore the consequences of an act before engaging in it; (3) (lack of) Perseverance, difficulty remaining focused on a boring and/or difficult task; and (4) Sensation seeking, the tendency to pursue new and exciting activities. Importantly, the factor structure of this UPPS impulsivity model has been confirmed in many subsequent studies, using multiple methodologies (Billieux et al., 2012).

Although the UPPS impulsivity facets have not been examined in relation to posttraumatic outcomes, their role has been demonstrated in other areas of general emotional distress. Recently, it has been shown in community samples that depressed or anxious moods correlate with urgency and lack of perseverance to a greater degree than with other impulsivity facets (Billieux et al., 2012; Smith, Guller, & Zapolski, 2013). It is reasonable to argue that this relationship is mediated by individual differences in cognitive processes, such as appraisal schemas or coping strategies. In accordance with this prediction, a study by d'Acremont and Van der Linden (2007) found that urgency and lack of perseverance indirectly explained depressive mood. This relation was mediated by the cognitive emotion regulation strategies assessed with the Cognitive Emotion Regulation Questionnaire (CERQ; Garnefski et al., 2001). The CERQ identifies nine emotion regulation strategies, five of which are considered appropriate (e.g., putting into perspective) and four of which are considered inappropriate (e.g., self-blame). The results indicated that individuals with high levels of urgency and lack of perseverance self-reported more severe depressive moods, possibly because they were more likely to use inappropriate emotion regulation strategies. Cognitive emotion regulation was therefore shown to be an important mediator in the relationship between impulsivity and depressive moods. Interestingly, research has not yet evaluated this relationship while taking both trauma exposure and the multifaceted construct of impulsivity into account.

The goal of the current study was to explore the paths between trauma, impulsivity, cognitive emotion regulation, and depressive mood. Our theoretical model of depressive mood was based on d'Acremont and Van der Linden's (2007) study, which found indirect relationships between two impulsivity facets (urgency and lack of perseverance) and depressive mood, mediated by adaptive and maladaptive cognitive emotion regulation strategies. Our model expands on these findings by accounting for the differential influence of impulsivity on emotion regulation and depressive mood as a function of previous lifetime trauma exposure.

## Method

### Participants

Ninety-three members of the Geneva community (55 female) voluntarily completed a packet of questionnaires presented in a fixed order. Participants were recruited through contacts made by three undergraduate psychology students who were asked to enroll fluent French-speaking adults willing to anonymously self-report on their emotions. Participants’ anonymity was guaranteed by having respondents fill in the scales on their own, and giving them the opportunity to return the questionnaires in a sealed envelope. Signed consent forms were stored separately. The study protocol was approved by the ethics committee of the Psychology Department of the University of Geneva.

Participants were 20 to 40 years old (*M* age=24.69; SD=5.03) with a mean of 5.68 years (SD=2.85, range*:* 0–14 years) of post-primary education. The sample was composed of 53 students (60.2%), 12 white-collar workers (13.6%), 20 blue-collar workers (22.7%), and 3 unemployed or retired individuals (3.4%). As assessed with a checklist, no respondent self-reported a history of mental disorders, including PTSD.

### Measures

#### Traumatic events checklist

The checklist portion of the Posttraumatic Diagnostic Scale (PDS-F; Hearn, Ceschi, Brillon, Fürst, & Van der Linden, 2012) presents 11 traumatic events (Table 1) and asks participants to state which event(s), if any, they have experienced during their lifetime. A 12th open question allows for the description of any other event not included in the checklist. In our sample, 38 participants (41%) did not report trauma exposure whereas 55 participants (59%) reported having experienced or witnessed at least one trauma during their lifetime. On average, our participants self-reported 1.20 traumatic events (SD=1.31). Previous literature on different community samples consistently showed that trauma is a ubiquitous phenomenon even in non-clinical individuals living in the developed world. In a representative community sample, for instance, Elhai et al. (2012) reported that 67% of college students endorsed at least one traumatic event based on a conventional PTSD trauma classification. Despite this very high prevalence of trauma, only a minority of the traumatized community individuals present clinical PTSD (about 0.4–14%, depending on the study). Thus, despite their relatively young mean age and the non-clinical nature of our participants, the trauma incidence we captured with the PDS-F checklist appears to be consistent with previous descriptions of traumatization in young adults in Western countries.

**Table 1:** Absolute frequency of trauma endorsed by PDS-F traumatic event [*n* of respondents (% with reference to the whole sample)]

Trauma	Endorsement
Serious accident	33 (35.5%)
Natural disaster	9 (9.7%)
Non-sexual assault by family member	11 (11.8%)
Non-sexual assault by stranger	19 (20.4%)
Sexual assault by family member	4 (4.3%)
Sexual assault by stranger	1 (1.1%)
Military combat	—
Sexual contact when younger than 18	12 (12.9%)
Imprisonment	2 (2.2%)
Torture	—
Life-threatening illness	3 (3.2%)
Other trauma	18 (19.4%)

*N*=93.

*N*_(0 trauma)_=38; *N*_(1 trauma)_=20; *N*_(2 traumas)_=21; *N*_(3 or more trauma)_=14.

Among the 59% of individuals reporting at least one traumatic experience, 20 individuals (21%) endorsed one traumatic event, 21 (23%) two traumatic events, and 14 (15%) three or more traumatic events. Table 1 shows the endorsement frequency by trauma.

Given the large number of participants who reported no traumatic events, in the present study the variance of traumatic exposure is evaluated as a dichotomous variable (+1 for exposure versus −1 for non-exposure). In preliminary statistical analyses, we compared the results obtained with trauma exposure as a dichotomous variable with those obtained with a continuum variable of trauma exposure (absolute frequency of trauma endorsed). As expected, the two variables strongly correlated (*r*(93)=0.77; *p*<0.001). The introduction of the continuum measure of trauma endorsement in our analyses did not change the general profile of statistical findings described below.

**Fig. 1:**
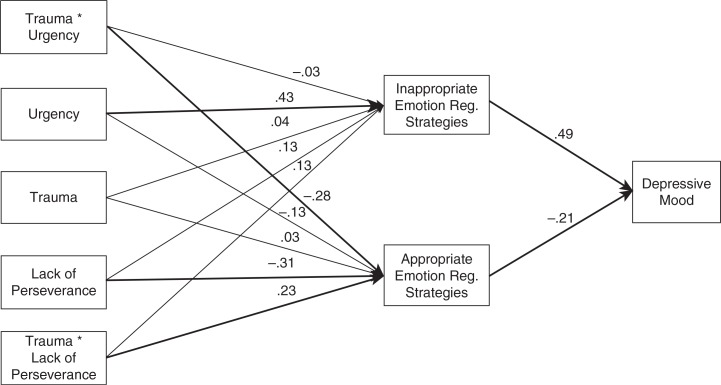
Trauma and impulsivity predicting emotion regulation, and mediation by emotion regulation for predicting depressive mood; χ^2^(6)=7.049, *p*=0.3164; RMSEA [90% confidence interval]=0.043 [0; 0.147] CFI=0.986; TLI=0.957; *N*=93. Bold arrows indicate significant standardized regression coefficients (*p*<0.05). Normal arrows indicate non-significant standardized regression coefficients.

In agreement with PDS instructions (Foa, Cashman, Jaycox, & Perry, 1997), only participants who reported at least one traumatic event assessed their PTSD symptoms on the second part of the PDS-F. Six out of the 55 participants (6% of the whole sample) presented all the criteria of clinically relevant PTSD. From an interindividual perspective, the severity of PTSD symptoms did not account for a significant amount of variance of depressive mood (*r*(52)=0.18, *ns*) or of cognitive emotion regulation strategies (*r*(52)=0.04, *ns*, and *r*(52)=0.05, *ns*, respectively, for appropriate and inappropriate strategies). Moreover, additional regression analyses indicate that the depressive mood model described in Fig. 1 remains stable even after controlling for the presence of PTSD and the severity of PTSD symptoms. Thus, severity of PTSD symptoms was not introduced into the final model.

#### UPPS Impulsive Behavior Scale (UPPS; Van der Linden et al., 2006)

The UPPS measures participants’ agreement with 45 items on a Likert scale ranging from 1 (totally agree) to 4 (totally disagree). Four different facets of impulsivity are assessed: *Urgency* (e.g., “When I am upset I often act without thinking”), *lack of Perseverance* (e.g., “I tend to give up easily”), *lack of Premeditation* (e.g., “I usually make up my mind through careful reasoning”), and *Sensation seeking* (e.g., “I welcome new and exciting experiences and sensations, even if they are a little frightening and unconventional”). The validation of the French version of the UPPS yielded a four-factor structure with good internal consistency (Cronbach's *α* ranging between 0.77 and 0.83 depending on subscale; Van der Linden et al., 2006). In the current sample, the UPPS showed very good internal consistency, with Cronbach's *α* ranging from 0.87 to 0.90 for the different dimensions (Table 2).

**Table 2:** Cronbach's α, means, standard deviations, and Pearson correlations for trauma, UPPS impulsivity facets, emotion regulation, and depressive mood

	*α*	Mean (SD)	Trauma^a^	Urgency	Lack persev.	Lack premed.	Sens. seek.	Depress. mood	Inapp. coping
Trauma^a^	–	0.59 (0.49)	–						
Urgency	0.88	27.71 (6.64)	0.09	–					
Lack of perseverance	0.87	20.59 (5.11)	0.02	0.36**	–				
Lack of premeditation	0.88	22.96 (5.33)	−0.01	0.45**	0.45**	–			
Sensation seeking	0.90	30.47 (8.59)	0.17	0.13	0.13	0.43**	–		
Depressive mood	0.84	4.32 (3.80)	0.12	0.41**	0.13	−0.01	−0.05	–	
Inappropriate coping	0.82	36.26 (8.12)	0.08	0.48**	0.32*	0.10	−0.07	0.51**	–
Appropriate coping	0.87	68.62 (12.26)	0.02	−0.31*	−0.30*	−0.05	0.14	−0.27*	−0.13

a Trauma exposure (+1=trauma exposure, –1=no exposure). *N=*93.

* p<0.01.

** p<0.001.

#### Cognitive Emotion Regulation Questionnaire (CERQ; Jermann, Van der Linden, d'Acremont, & Zermatten, 2006)

The CERQ is a 36-item self-report questionnaire evaluating participants’ use of nine different cognitive coping strategies. A Likert scale from 1 (almost never) to 5 (almost always) assesses participants’ agreement with each self-statement. The nine strategies relate to two subgroups of cognitive emotion regulation: inappropriate and appropriate. Appropriate strategies include *acceptance* (e.g., “I think that I must learn to live with it”), *positive refocusing* (e.g., “I think of nicer things than what I have experienced”), *refocus on planning* (e.g., “I think about how I can best cope with the situation”), *positive reappraisal* (e.g., “I think I can learn something from the situation”), and *putting into perspective* (e.g., “I tell myself that there are worse things in life”). Inappropriate strategies include *self-blame* (e.g., “I think that basically the cause must lie within myself”), *rumination* (e.g., “I dwell upon the feelings the situation has evoked in me”), *catastrophizing* (e.g., “I continually think how horrible the situation has been”), and *blaming others* (e.g., “I feel that others are responsible for what has happened”). The French validation of the CERQ reproduced (1) the nine-factor structure of the original English version of the scale and (2) the two-factor structure for inappropriate and appropriate strategies (Jermann et al., 2006). In Jermann et al. (2006) and in our sample, Cronbach's *α* were, respectively, 0.89 and 0.87 for appropriate strategies, and 0.82 and 0.82 for inappropriate strategies (Table 2).

#### Short Depression-Happiness Scale (SDHS; Joseph, Linley, Harwood, Lewis, & McCollam, 2004)

The SDHS is a reliable measure of depressive mood described on a depression-happiness continuum. Participants are asked to judge their feelings with reference to six items representing depression (e.g., “I felt that life was meaningless”) and happiness (e.g., “I felt happy”) and report their answers on four-point Likert scales ranging from 0 (never) to 3 (often). The SDHS presents a unidimensional factor structure (high scores represent happiness) and a good internal consistency, with a Cronbach's *α* of 0.77 to 0.92 and 0.84, respectively, across previous studies (Joseph et al., 2004) and in the current sample (Table 2). The SDHS has been demonstrated to have good test–retest stability over a 2-week interval (*r*=0.68, *p*<0.001), good convergent validity with other well-established measures of happiness and depression, such as the Beck Depression Inventory (BDI; Beck, Steer, & Brown, 1996; *r*=0.63−*r*=0.68, *p*<0.001), and good discriminant validity with anxiety and hysteria.

### Statistical analysis

Path analysis was performed using MPlus5 (Muthén & Muthén, 2006). Interactions (trauma with urgency, trauma with lack of perseverance) were calculated to predict appropriate and inappropriate emotion regulation. Urgency and lack of perseverance were centered in order to reduce potential multicollinearity between the main and interaction effects (Cohen, Cohen, West, & Aiken, 2003). The mediation analyses evaluated whether the strongest relationships between independent (urgency, lack of perseverance, interactions) and dependent (depressive mood) variables were accounted for by mediators (appropriate and inappropriate emotion regulation). The indirect relationships through the mediators should remain strong when direct paths between the independent and dependent variables are also accounted for in the model. MPlus calculates mediation by the product of coefficient strategy (Preacher, Rucker, & Hayes, 2007). Model fit was assessed using the chi-square (*χ* ^2^), Root Mean Square Error of Approximation (RMSEA), Comparative Fit Index (CFI), and Tucker-Lewis Index (TLI). A non-significant *χ* ^2^ indicates a good fit (Byrne, 1994). CFI and TLI greater than 0.90 indicate a good fit, whereas an RMSEA below 0.05 shows a close fit between the model and the sample data per degree of freedom (Hu & Bentler, 1999).

## Results

Means, standard deviations, and Pearson correlations for trauma, impulsivity, cognitive emotion regulation, and depressive mood are presented in Table 2.

Figure 1 displays the path model that had the closest fit to the data: *χ* ^2^(6)=7.049, *p*=0.316; RMSEA [90% confidence interval]=0.043 [0; 0.147], CFI=0.986, TLI=0.957. Our first aim was to evaluate the relationship between trauma and emotion regulation and depressive mood, and the mediating role of emotion regulation. Trauma did not significantly predict emotion regulation, either for inappropriate (*b*=0.04, *p*=0.634) or for appropriate strategies (*b*=0.03, *p*=0.725); therefore, mediation by emotion regulation could not be confirmed. However, the regressions of depressive mood on inappropriate (*b*=0.49, *p*<0.001) and appropriate strategies (*b*=0.21, *p*=0.016) were significant.

**Fig. 2:**
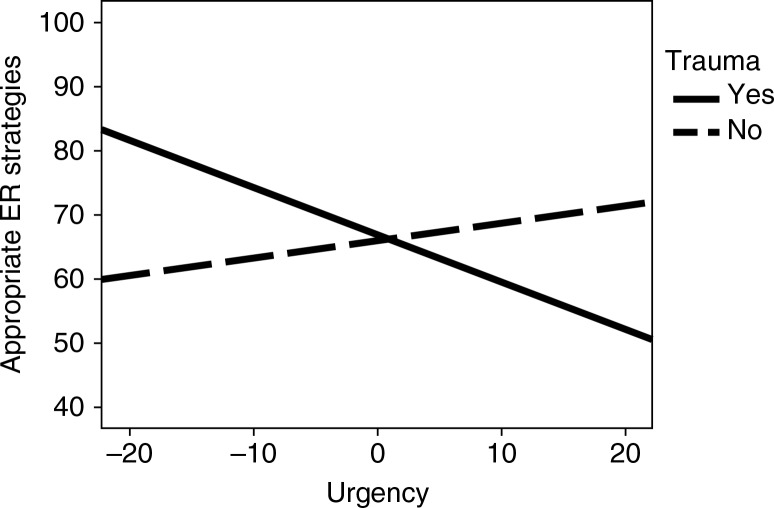
Interaction effect between trauma and urgency on appropriate emotion regulation (ER) strategies.

Our second aim was to investigate whether urgency and lack of perseverance correlated with emotion regulation and depressive mood. Concerning urgency, a significant effect was revealed for inappropriate emotion regulation (*b*=0.43, *p*<0.001), whereas a non-significant effect was found for appropriate emotion regulation (*b*=0.13, *p*=0.232). Mediation by inappropriate emotion regulation was confirmed (*b*=0.21, *p*<0.001). As for the moderation of trauma in the relationship between urgency and emotion regulation, a significant interaction effect was found for appropriate emotion regulation (*b*=0.28, *p*=0.008) and a non-significant interaction effect was found for inappropriate emotion regulation (*b*=0.03, *p*=0.764). Figure 2 illustrates the moderation of trauma in the relationship between urgency and emotion regulation. The lowest level of appropriate emotion regulation strategies is observed in participants who reported a high score for urgency in conjunction with previous exposure to trauma. The mediating role of appropriate strategies in the relationship between trauma and urgency (interaction) and depressive mood revealed a tendency toward significant mediation (*b*=0.06, *p*=0.078).

**Fig. 3:**
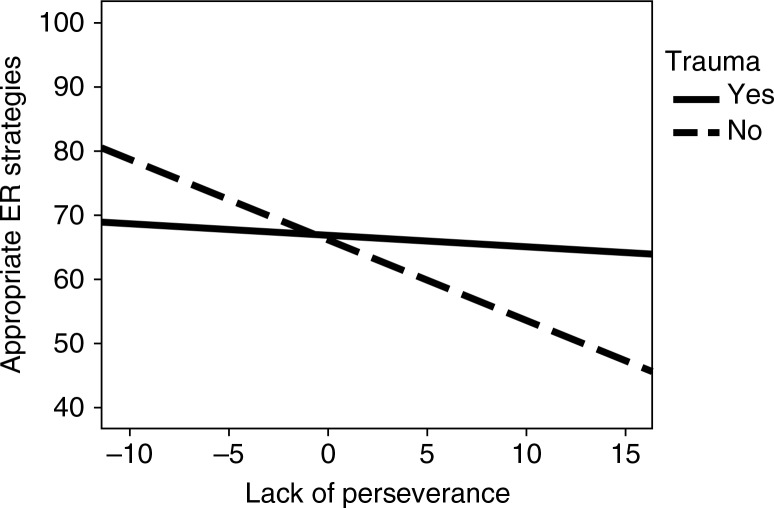
Interaction effect between trauma and lack of perseverance on appropriate emotion regulation (ER) strategies.

The results showed a negative path between lack of perseverance and appropriate emotion regulation (*b*=0.31, *p*=0.002) and a non-significant path between lack of perseverance and inappropriate emotion regulation (*b*=0.13, *p*=0.198). The mediating role of appropriate strategies in the relationship between lack of perseverance and depressive mood revealed a tendency toward significance (*b*=0.07, *p*=0.063). Interaction effects between trauma and lack of perseverance showed a non-significant path for inappropriate emotion regulation (*b*=0.13, *p*=0.205) and a significant path for appropriate emotion regulation (*b*=0.23, *p*=0.029). Figure 3 illustrates the moderation of trauma in the relationship between lack of perseverance and emotion regulation. Interestingly, trauma-exposed participants with higher lack of perseverance dispositions reported more appropriate emotion regulation strategies than other participants. Finally, mediation by appropriate strategies of the relationship between trauma and lack of perseverance (interaction) and depression was non-significant (*b*=0.05, *p*=0.109).

A second model assessed the same indirect paths examined in the original model while also examining the direct paths between the independent variables (trauma, urgency, lack of perseverance, interactions) and depressive mood. None of the direct links to depressive mood were significant: trauma exposure (*b*=0.07, *p*=0.386), urgency (*b*=0.15, *p*=0.159), lack of perseverance (*b*=0.10, *p*=0.321), interaction between trauma and urgency (*b*=0.10, *p*=0.335), and interaction between trauma and lack of perseverance (*b*=0.10, *p*=0.327). The indirect effects via emotion regulation observed in the original model remained significant in this second model. Chi-square model comparison showed that the overall fit of this second model was not significantly better than the original one, Δ*χ* ^2^ (5)=6.886, *p*=0.246. Therefore, the more parsimonious structure of the original model should be preferred.

## Discussion

The results of the current study add to a very small body of literature on the effects of trauma exposure on impulsivity, cognitive emotion regulation, and depressive mood. Consistent with previous studies, urgency and lack of perseverance were found to be the facets of impulsivity associated with depressive mood (Billieux et al., 2012; Smith et al., 2013). Likewise, in accordance with d'Acremont and Van der Linden (2007), these relationships were found to be mediated by cognitive emotion regulation strategies: Although urgency was associated with a tendency to rely on more inappropriate regulation strategies, lack of perseverance was associated with a tendency to rely on more appropriate ones. As such, these findings corroborate the view that impulsive traits influence emotional reactions via an indirect effect on coping strategies.

Our study is the first to add to this impulsivity–depressive mood model, the potential unshared environmental effect of previous exposure to trauma. In our study, trauma exposure did not significantly predict appropriate or inappropriate cognitive emotion regulation strategies *per se*. However, it provided evidence that trauma exposure, interacting with urgency and lack of perseverance, predicted emotion regulation above and beyond all the other variables. These trauma–impulsivity interactions corresponded with specific patterns of depressive mood expression. In the context of previous traumatic experiences, the individuals at greatest risk of reporting severe negative feelings are those who simultaneously report high scores for urgency and perseverance. These results suggest that the endophenotypical expression of impulsive dispositions, such as urgency and lack of perseverance, might actually be modulated by unshared environmental factors encountered during the lifetime.

Three main points arising from these findings warrant further discussion. First, in our study, trauma exposure did not have a direct effect on cognitive emotion regulation and depressive mood. Thus, our data could not confirm the mediating role of emotion regulation in the relationship between trauma and depression observed elsewhere (Raes & Hermans, 2008). Conceivably, our measure of lifetime trauma endorsement was not sufficiently sensitive to capture interindividual differences in traumatization in our community sample. On the contrary, Raes and Hermans (2008) used an indicators of trauma exposure based on self-relevant appraisals of severity of childhood traumatic events.

Additional studies considering differences in trauma types (e.g., in adulthood versus in childhood; recurrent versus single event) and trauma measures (more subjective, such as trauma appraisals, or more objective, such as military action records) would certainly shed new light on our understanding of the differential expression of personal dispositions as a function of different types of unshared environmental factors.

Contrary to our findings, previous work done on a comparable community sample did describe a moderate association between depression and trauma exposure measured with the PDS-F (David et al., 2008). Interestingly, this study found that trauma was associated with the affective-cognitive symptoms of the BDI (e.g., sadness, self-criticism) but not with the somatic symptoms of that scale (e.g., fatigue, sleep disturbance). Additional research is thus needed to clarify the impact of trauma on different facets of the depressive phenomenon.

Second, as expected, urgency was significantly correlated with inappropriate cognitive emotion regulation strategies that mediated the relationship between urgency and depressive mood. In accordance with d'Acremont and Van der Linden's (2007), this suggests that adults who reported high scores for urgency were more depressed, possibly because they tended to implement inappropriate coping strategies. In addition, in our study, urgency showed an inverse correlation with appropriate strategies. Specific inhibition mechanisms underlying impulsivity traits may explain these results. Urgency is indeed related to difficulties deliberately suppressing dominant or automatic responses (Gay, d'Acremont, Rochat, Billieux, & Van der Linden, 2008). Emotion regulation also depends on inhibitory functions to shift the focus away from negative emotional information and achieve the desired emotional response (Ochsner & Gross, 2005). Presumably, reappraisal and other appropriate cognitive coping strategies cannot be successfully applied when one's attention is bogged down in negative emotions, as it is during ruminative thinking for example (Troy & Mauss, 2011). Our findings suggest that individuals who score high for urgency tend to show an internally focused coping style, as in self-blame, rumination, and catastrophizing. This coping style may be understood as reflecting a difficulty in inhibiting the use of inappropriate coping strategies that alleviate negative affect in the short term but maintain it in the long run.

We were particularly interested in the interaction between trauma and urgency and its relationship with cognitive emotion regulation. Our findings showed that urgency made it harder for trauma-exposed individuals to use appropriate strategies. Thus, even though, in general, urgency may be related to a strong tendency to rely on internally focused emotion regulation strategies (e.g., rumination and self-blaming), when one specifically controls for trauma exposure, urgency may be associated with reduced reliance upon the appropriate coping strategies usually associated with posttraumatic growth, such as reappraisal, perspective-taking, and acceptance. This suggests that individuals who report trauma may have insufficient cognitive resources available to take the extra step toward implementing appropriate strategies normally observed in non-traumatized persons. Indeed, trauma exposure and the intrusive memories arising from trauma have been associated with poor working memory performance (Klein & Boals, 2001). Trauma exposure may therefore reduce attention and inhibit the cognitive resources needed for reappraisal (Troy & Mauss, 2011). Clearly, the executive processes that underlie both urgency and emotion regulation must be further studied in trauma-exposed individuals before these tentative interpretations can be incorporated into an integrated cognitive diathesis-stress view of depressive mood.

Third, in agreement with d'Acremont and Van der Linden (2007), lack of perseverance was associated with less frequent reliance upon appropriate coping strategies. Recent studies have shown that lack of perseverance may be associated with difficulties inhibiting intrusions and focusing on a task when presented with familiar but irrelevant information (Gay et al., 2008). This mechanism may be particularly relevant in the case of trauma exposure, which can lead to recurrent intrusive thoughts and images, even in non-clinical populations (Rubin, Boals, & Berntsen, 2008). Taking both factors into consideration, it can be postulated that recurrent involuntary posttraumatic symptoms, such as intrusions, may disrupt the use of appropriate coping strategies by continually activating out-of-date information and not allowing the individual to develop new perspectives.

Unexpectedly, our study showed that, for trauma-exposed individuals, the use of appropriate emotion regulation strategies was qualified by perseverance disposition. Contrary to our predictions, however, our findings suggested that lack of perseverance may actually encourage (rather than disrupt) effective emotion regulation after trauma exposure.

Although we do not discount the established viewpoint that lack of perseverance is generally associated with negative consequences for emotion regulation, it is still possible to imagine that there may be positive consequences as well. On the contrary, perseverance addresses an individual's desire to see projects through to the end—not to give up on something they have started—and an ability to concentrate and finish tasks. In some contexts, such as rumination (a state of pervasive and repetitive self-focused thinking dwelling on negative thoughts and images that promote depression (Nolen-Hoeksema,^1987^) and PTSD (Ehring, Frank, & Ehlers, 2008), perseverant individuals may resort to rumination because they think that bringing closure to a negative event requires a profound understanding of what happened. In some cases, this profound understanding may be similar to ruminative thinking and, paradoxically, may stir up negative affect.

However, lack of perseverance refers to an individual's tendency to ignore little tasks when there are too many to handle, giving up on projects and not seeing projects through to the end. In some situations, these behaviors may be related to distraction. Although research asserts that the long-term emotional consequences of distraction are unstable, recent studies have come to a consensus that distraction does indeed have short-term benefits (Troy & Mauss, 2011). In accordance with this idea, individuals who score high for lack of perseverance may become distracted because they are less interested in questioning and delving into the circumstances underlying a terrible event. They may reduce their negative affect simply by engaging in different activities. Given the extraordinary circumstances of traumatic life experiences, perseverant thinking about highly emotional content may be toxic and counter-productive. It is possible that non-perseverative thinking, such as distraction, is advantageous after a traumatic event, because it directs attention away from negative affect. Considering the doubts in the literature regarding the long-term benefits of distraction, future studies should strive to determine whether appropriate emotion regulation is maintained as the time elapsed since the trauma increases. Clearly, this unexpected interaction effect needs to be replicated before any explanations of it can be considered to be other than tentative. Nonetheless, the current study supports the multifaceted conceptualization of impulsivity by showing that urgency and lack of perseverance differentially influence emotional outcomes after trauma. Moreover, our findings caution against the tendency to systematically regard impulsivity as maladaptive.

Several limitations on the study must be mentioned. First, the cross-sectional design of this study limits the interpretation of mediation in terms of causality. Second, depressive mood was assessed with the SDHS. Other aspects of cognitive, physiological, and affective phenomena of depression and general distress could have been considered and might have led to different conclusions. Third, our trauma exposure measure was limited to a dichotomous variable derived from the PDS-F checklist. Our study should be replicated with more subtle indicators of traumatic exposure, possibly allowing distinctions, for instance, between early versus late trauma onset, objective versus subjective trauma impact, and recurrent versus single trauma occurrence. These distinctions appear to be extremely important in understanding the effect of unshared environmental factors on cognitive emotion regulation. Fourth, in agreement with the PDS-F instructions, PTSD symptom severity was assessed only for the 59% of the sample who endorsed at least one traumatic event. Although our preliminary statistical analyses strongly suggest that PTSD severity cannot account for our findings, a more general measure of PTSD severity for non-clinical individuals might be instrumental in drawing conclusions on posttraumatic outcomes.

In spite of these limitations, the current study demonstrated that trauma and impulsivity affect the cognitive emotion regulation strategies associated with depressive mood. Trauma exposure did not directly influence emotion regulation or depressive mood *per se*, whereas urgency and lack of perseverance proved to have consequences for emotion regulation. In the context of trauma, urgency hampered emotion regulation whereas lack of perseverance unexpectedly encouraged appropriate emotion regulation. The mechanisms responsible for the differential impact of trauma and impulsivity on emotional outcomes are worthy of future investigation.

## References

[CIT0001] Aidman E. V, Kollaras-Mitsinikos L (2006). Personality dispositions in the prediction of posttraumatic stress reactions. Psychological Reports.

[CIT0002] Amone-P'Olak K, Garnefski N, Kraaij V (2007). Adolescents caught between fires: Cognitive emotion regulation in response to war experiences in Northern Uganda. Journal of Adolescence.

[CIT0003] 
Beck A. T, Steer R. A, Brown G. K (1996). Manual for the Beck Depression Inventory—II.

[CIT0004] Ben-Zur H, Zeidner M (2009). Threat to life and risk-taking behaviors: A review of empirical findings and explanatory models. Personality and Social Psychology Review.

[CIT0005] Bevilacqua L, Carli V, Sarchiapone M, George D. K, Goldman D, Roy A, Enoch M. A (2012). Interaction between FKBP5 and childhood trauma and risk of aggressive behavior. Archives of General Psychiatry.

[CIT0006] Billieux J, Gay P, Rochat L, Van der Linden M (2010). The role of urgency and its underlying psychological mechanisms in problematic behaviours. Behaviour Research and Therapy.

[CIT0007] Billieux J, Rochat L, Ceschi G, Carré A, Offerlin-Meyer I, Defeldre A.-C (2012). Validation of a short French version of the UPPS-P Impulsive Behavior Scale. Comprehensive Psychiatry.

[CIT0008] Braquehais M. D, Oquendo M. A, Baca-García E, Sher L (2010). Is impulsivity a link between childhood abuse and suicide?. Comprehensive Psychiatry.

[CIT0009] Brewin C. R (2003). Post-traumatic stress disorder: Malady or myth?.

[CIT0010] Brodsky B. S, Oquendo M, Ellis S. P, Haas G. L, Malone K. M, Mann J. J (2001). The relationship of childhood abuse to impulsivity and suicidal behavior in adults with major depression. American Journal of Psychiatry.

[CIT0011] Byrne B. M (1994). Structural equation modeling with EQS and EQS/Windows.

[CIT0012] Carli V, Jovanović N, Podlešek A, Roy A, Rihmer Z, Maggi S (2010). The role of impulsivity in self-mutilators, suicide ideators and suicide attempters—A study of 1265 male incarcerated individuals. Journal of Affective Disorders.

[CIT0013] Ceschi G, Hearn M, Billieux J, Van der Linden M (2011). Lifetime exposure to adverse events and reinforcement sensitivity in obsessive-compulsive prone individuals. Behaviour Change.

[CIT0014] Cohen J, Cohen P, West S. G, Aiken L. S (2003). Applied multiple regression/correlation analysis for the behavioral sciences.

[CIT0015] d'Acremont M, Van der Linden M (2007). How is impulsivity related to depression in adolescence? Evidence from a French validation of the cognitive emotion regulation questionnaire. Journal of Adolescence.

[CIT0016] David M, Ceschi G, Billieux J, Van der Linden M (2008). Depressive symptoms after trauma: Is self-esteem a mediating factor?. The Journal of Nervous and Mental Disease.

[CIT0017] Ehring T, Frank S, Ehlers A (2008). The role of rumination and reduced concreteness in the maintenance of posttraumatic stress disorder and depression following trauma. Cognitive Therapy Research.

[CIT0018] Elhai J. D, Miller M. E, Ford J. D, Biehn T. L, Palmieri P. A, Frueh B. C (2012). Posttraumatic stress disorder in DSM-5: Estimates of prevalence and symptom structure in a nonclinical sample of college students. Journal of Anxiety Disorders.

[CIT0019] Fehon D. C, Grilo C. M, Lipschitz D. S (2005). A comparison of adolescent inpatients with and without a history of violence perpetration. Journal of Nervous and Mental Disease.

[CIT0020] Foa E. B, Cashman L, Jaycox L, Perry K (1997). The validation of a self-report measure of posttraumatic stress disorder: The Posttraumatic Diagnostic Scale. Psychological Assessment.

[CIT0021] Garnefski N, Koopman H, Kraaij V, ten Cate R (2009). Cognitive emotion regulation strategies and psychological adjustment in adolescents with a chronic disease. Journal of Adolescence.

[CIT0022] Garnefski N, Kraaij V (2006). Relationship between cognitive emotion regulation strategies and depressive symptoms: A comparative study of five specific samples. Personality and Individual Differences.

[CIT0023] Garnefski N, Kraaij V, Spinhoven P (2001). Negative life events, cognitive emotion regulation and emotional problems. Personality and Individual Differences.

[CIT0024] Garnefski N, Teerds J, Kraaij V, Legerstee J, van den Kommer T (2004). Cognitive emotion regulation strategies and depressive symptoms: Differences between males and females. Personality and Individual Differences.

[CIT0025] Gay P, d'Acremont M, Rochat L, Billieux J, Van der Linden M (2008). Heterogeneous inhibition processes involved in different facets of self-reported impulsivity: Evidence from a community sample. Acta Psychologica.

[CIT0026] Hearn M, Ceschi G, Brillon P, Fürst G, Van der Linden M (2012). A French adaptation of the Posttraumatic Diagnostic Scale. Canadian Journal of Behavioural Science.

[CIT0027] Hu L.-T, Bentler P. M (1999). Cutoff criteria for fit indexes in covariance structure analysis: Conventional criteria versus new alternatives. Structural Equation Modeling.

[CIT0028] Jallade C, Sarfati Y, Hardy-Baylé M.-C (2005). Clinical evolution after self-induced or accidental traumatism: A controlled study of the extent and the specificity of suicidal catharsis. Journal of Affective Disorders.

[CIT0029] Jermann F, Van der Linden M, d'Acremont M, Zermatten A (2006). Cognitive Emotion Regulation Questionnaire (CERQ): Confirmatory factor analysis and psychometric properties of the French translation. European Journal of Psychological Assessment.

[CIT0030] Joseph S, Dalgleish T, Thrasher S, Yule W (1997). Impulsivity and post-traumatic stress. Personality and Individual Differences.

[CIT0031] Joseph S, Linley P. A, Harwood J, Lewis C. A, McCollam P (2004). Rapid assessment of well-being: The Short Depression-Happiness Scale (SDHS). Psychology and Psychotherapy: Theory, Research and Practice.

[CIT0032] Klein K, Boals A (2001). The relationship of life event stress and working memory capacity. Applied Cognitive Psychology.

[CIT0033] Kraaij V, Arensman E, Garnefski N, Kremers I (2007). The role of cognitive coping in female victims of stalking. Journal of Interpersonal Violence.

[CIT0034] 
Martin R. C, Dahlen E. R (2005). Cognitive emotion regulation in the prediction of depression, anxiety, stress and anger. Personality and Individual Differences.

[CIT0035] Muthén L. K, Muthén B. O (2006). Mplus user's guide.

[CIT0036] Nolen-Hoeksema S (1987). Sex differences in unipolar depression: Evidence and theory. Psychological Bulletin.

[CIT0037] Ochsner K. N, Gross J. J (2005). The cognitive control of emotion. Trends in Cognitive Sciences.

[CIT0038] Preacher K. J, Rucker D. D, Hayes A. F (2007). Addressing moderated mediation hypotheses: Theory, methods, and prescriptions. Multivariate Behavioral Research.

[CIT0039] Raes F, Hermans D (2008). On the mediating role of subtypes of rumination in the relationship between childhood emotional abuse and depressed mood: Brooding versus reflection. Depression and Anxiety.

[CIT0040] Rubin D. C, Boals A, Berntsen D (2008). Memory in posttraumatic stress disorder: Properties of voluntary and involuntary, traumatic and nontraumatic autobiographical memories in people with and without posttraumatic stress disorder symptoms. Journal of Experimental Psychology: General.

[CIT0041] Schwartz D, Proctor L. J (2000). Community violence exposure and children's social adjustment in the school peer group: The mediating roles of emotion regulation and social cognition. Journal of Consulting and Clinical Psychology.

[CIT0042] Simmen-Janevska K, Brandstätter V, Maercker A (2012). The overlooked relationship between motivational abilities and posttraumatic stress: A review. European Journal of Psychotraumatology.

[CIT0043] Smith G. T, Guller L, Zapolski T. C (2013). A comparison of two models of urgency: Urgency predicts both rash action and depression in youth. Clinical Psychological Science.

[CIT0044] Troy A, Mauss I. B, Southwick S, Charney D, Friedman M, Litz B (2011). Resilience in the face of stress: Emotion regulation as a protective factor. Resilience to stress.

[CIT0045] Tull M, Barrett H. M, McMillan E. S, Roemer L (2007). A preliminary investigation of the relationship between emotion regulation difficulties and posttraumatic stress symptoms. Behavior Therapy.

[CIT0046] Van der Linden M, d'Acremont M, Zermatten A, Jermann F, Larøi F, Willems S (2006). A French adaptation of the UPPS Impulsive Behavior Scale: Confirmatory factor analysis in a sample of undergraduate students. European Journal of Psychological Assessment.

[CIT0047] Whiteside S. P, Lynam D. R (2001). The five factor model and impulsivity: Using a structural model of personality to understand impulsivity. Personality and Individual Differences.

